# Preliminary Feasibility and Acceptability of a Cognitive Behavioral Therapy Combining Group and Individual Sessions for Obsessive–Compulsive Disorder in Clinical Practice

**DOI:** 10.3390/bs16040529

**Published:** 2026-04-01

**Authors:** Yasue Mitamura, Toshitaka Hamamura, Koki Haruguchi, Fumi Imamura, Shinsuke Kito, Hironori Kuga

**Affiliations:** 1 National Center for Cognitive Behavior Therapy and Research, National Center of Neurology and Psychiatry, 4-1-1 Ogawa Higashi, Kodaira-shi, Tokyo 187-8511, Japan; ymitamura@ncnp.go.jp (Y.M.); thamamura@ncnp.go.jp (T.H.); 2 Brain Bioregulatory Science, Cooperative Graduate School, Jikei University Graduate School of Medicine, 3-25-8 Nishi-Shimbashi, Minato-ku, Tokyo 105-8461, Japan; 3 Department of Clinical Psychology, National Center of Neurology and Psychiatry, 4-1-1 Ogawa Higashi, Kodaira-shi, Tokyo 187-8511, Japan; koki.haruguchi@mountsinai.org (K.H.); fimamura@ncnp.go.jp (F.I.); 4 Department of Psychiatry, Mount Sinai Behavioral Health Center, Icahn School of Medicine at Mount Sinai, 45 Rivington St, New York, NY 10002, USA; 5 Department of Psychiatry, National Center of Neurology and Psychiatry, 4-1-1 Ogawa Higashi, Kodaira-shi, Tokyo 187-8511, Japan; kito@ncnp.go.jp; 6 Department of Psychiatry, Jikei University of Medicine, 3-25-8 Nishi-Shimbashi, Minato-ku, Tokyo 105-8461, Japan

**Keywords:** obsessive–compulsive disorder, exposure response and prevention, group cognitive behavioral therapy

## Abstract

Hybrid cognitive behavioral therapy (CBT), combining group and individual sessions, for treating obsessive–compulsive disorder (OCD) has rarely been examined in routine clinical practice. This prospective observational study preliminarily evaluated the feasibility and acceptability of a hybrid CBT program implemented in Japan. The program consisted of one pre-treatment individual session, eight group sessions, and one post-treatment individual session. Feasibility and acceptability were assessed using dropout rates and written questionnaire feedback. Twenty-eight individuals (mean age = 36.1 ± 14.0 years) participated, with two dropouts. Seven participants reported that the program duration was too short, whereas the remaining participants considered it appropriate. Nineteen participants indicated their willingness to participate in a similar program. Open-ended feedback highlighted the importance of group composition and program content. Self-Rating Yale–Brown Obsessive Compulsive Scale scores decreased at Session 8 (estimate = −2.74, *p* = 0.002) and post-treatment (estimate = −4.16, *p* < 0.001) according to a linear mixed-effects model. Reductions were also observed in Sheehan Disability Scale, State–Trait Anxiety Inventory, and Clinical Global Impressions Scale scores, whereas Center for Epidemiologic Studies Depression Scale scores showed no significant change. These findings suggest the feasibility and acceptability of the program and may inform future program development.

## 1. Introduction

Obsessive–compulsive disorder (OCD) is a chronic and progressive mental illness with a lifetime prevalence of 1–3%. According to the World Health Organization, OCD is among the top 10 most disabling medical conditions worldwide (Murray et al., 1996). Persistence and worsening symptoms can severely impair social and occupational functioning. Moreover, individuals with OCD can experience profound social isolation (Friedman-Ezra et al., 2024; Timpano et al., 2014). As the disorder progresses, insights into the excessive nature of compulsive behaviors tend to diminish, thereby contributing to treatment resistance.

International guidelines recommend cognitive behavioral therapy (CBT), particularly exposure and response prevention (ERP), as a first-line treatment for OCD, alongside pharmacotherapy (American Psychiatric Association, 2007; Bandelow et al., 2023; National Institute for Health and Care Excellence, 2005). CBT targets cognitive distortions and maladaptive behavioral patterns, aiming to disrupt the cycle of anxiety-provoking thoughts, followed by avoidance or ritualistic behaviors. Its therapeutic benefits have been shown to persist beyond the end of treatment (Elsner et al., 2020). For example, Cabedo et al. (2018) reported that 58.6% of patients with OCD who received individual CBT achieved recovery, with sustained effects even 10 years after treatment.

Despite its effectiveness, the use of CBT in the treatment of OCD remains limited. Only 10–30% of individuals with OCD receive CBT, indicating substantial gaps in treatment accessibility (Schwartz et al., 2013). Provider-related factors include a lack of therapists, need for additional training, and concerns about exposure therapies (Farrell & Deacon, 2013; Mukai et al., 2025). Patient-related factors, such as treatment costs, insurance coverage status, feelings of shame, and doubts about treatment effectiveness have also been reported as barriers to treatment (Gellatly et al., 2017). Even if CBT is initiated, lack of insight, avoidance behaviors, and insufficient readiness for change can weaken therapeutic outcomes and make treatment continuation difficult (Visser et al., 2017; Wheaton et al., 2018).

Group CBT (CBGT) has been proposed as a promising alternative that allows simultaneous intervention for multiple patients and potentially addresses the shortage of trained therapists. In addition to logistical advantages, CBGT may foster peer support, role modeling, and enhanced self-understanding among participants (Kobak et al., 1995). Group therapy is also characterized by several therapeutic factors, such as instillation of hope, universality, altruism, interpersonal learning, and group cohesion, which facilitate psychological change within the group (Yalom & Leszcz, 2020). Meta-analyses and systematic reviews have supported its efficacy in treating OCD (Jonsson & Hougaard, 2009; Pozza & Dettore, 2017) and the National Institute for Health and Care Excellence (NICE) guidelines recommend CBGT for mild cases or those who desire low-intensity psychotherapy (National Institute for Health and Care Excellence, 2005). Although relatively few in number, previous studies have reported the effectiveness of CBGT for OCD (Kearns et al., 2010). Even the CBGT is equivalent to or slightly less effective than individual CBT in terms of efficacy, but it has equivalent acceptability (Pozza & Dettore, 2017; Wang et al., 2026).

Nevertheless, several issues related to the use of CBGT in OCD have been identified. One challenge lies in the group format, which makes it difficult to adequately conceptualize individual cases and tailor treatment for each patient’s clinical presentation. Given the substantial heterogeneity of OCD symptom dimensions, previous studies have emphasized that exposure hierarchies for contamination, checking, symmetry, and taboo obsessions differ markedly, underscoring the importance of individualized assessments before implementing CBGT (Abramowitz, 2006; Gillihan et al., 2012).

Several reports on hybrid forms of CBGT have been published internationally. Taylor and Reeder (2015) reported a case study describing combined intensive individual sessions and group sessions. Kunnari et al. (2024) conducted a feasibility study of a hybrid CBT program for adolescents with OCD which consisted of eight individual sessions, eight group sessions, and two sessions with parents.

These hybrid approaches may improve treatment accessibility while also compensating for the lack of individualized assessment in group CBT. However, the study by Kunnari et al. (2024) focused on adolescents, and the generalizability of their findings to adults remains unclear. As Taylor and Reeder’s (2015) report was a case study, outcomes for other group members were not reported. Moreover, intensive individual therapy delivered twice weekly requires substantial therapist commitment. Overall, there is limited evidence regarding the feasibility and acceptability of hybrid CBT programs for adults with OCD implemented in routine clinical settings.

In Japan, CBT for OCD is covered by the national health insurance system for up to 16 sessions (Igakutushinsha, 2025). However, in routine clinical practice some patients require additional sessions, and providing extended treatment can be difficult within the current insurance framework. Furthermore, until fiscal year 2025, insurance-reimbursed CBT has been limited to physicians and nurses working under physician supervision. Because physicians are often heavily engaged in outpatient and inpatient clinical duties, this restriction has limited the dissemination of CBT in Japan.

In contrast, outpatient group psychotherapy can be delivered by physicians together along with either psychiatric social workers or licensed psychologists, and it may be provided for up to six months (Igakutushinsha, 2025). Considering these practical and institutional conditions, we implemented a hybrid CBT program combining individual and group sessions conducted weekly as part of outpatient group psychotherapy. The program met the minimum treatment duration recommended in the NICE guidelines.

Hence, this study aimed to examine the feasibility and acceptability of a hybrid CBT program for OCD implemented in routine clinical practice. Its feasibility was evaluated using the dropout rates. Additionally, written questionnaire responses providing feedback on the group sessions were used to assess the program’s feasibility and acceptability. Thereafter, descriptive themes were derived from the open-ended questionnaire responses and analyzed to further explore participants’ experiences with the program. To provide exploratory insights into its potential therapeutic effects, preliminary changes in obsessive–compulsive, depressive, and anxiety symptoms were also examined before and after the program. Given the limited implementation of CBT for OCD in Japan, examining the feasibility and acceptability of this hybrid CBT program in routine clinical practice can inform future research on its effectiveness and guide improvements in its implementation.

## 2. Materials and Methods

### 2.1. Program Setting and Study Design

This program was conducted at the outpatient unit of a national center hospital that specializes in neurology and psychiatry in the suburbs of Tokyo, Japan. This hospital, as a national institution, provides one of the largest numbers of psychosocial treatments in the country. The development and implementation of this program stemmed from the need to address individuals who did not respond to individual CBT or experienced relapse.

Recruitment of this study occurred between June 2021 and March 2024. Informed consent was obtained from patients with OCD who expressed willingness to participate in the program and met the following eligibility criteria: (1) 18–65 years of age and (2) sufficient capacity to understand the informed consent procedure. Although no formal exclusion criteria were predefined in this study, participation in the program was determined through routine clinical procedures. Patients were referred by treating clinicians, and their suitability for participation was reviewed in meetings of the Department of Clinical Psychology in the hospital. Only those considered appropriate for group CBT based on clinical judgment (including safety considerations) were referred to the program. Ethical approval was obtained from the Institutional Review Board of the National Center of Neurology and Psychiatry (approval number: A2021-025). This study was registered in the University Hospital Medical Information Network (UMIN) Clinical Trials Registry (UMIN000044832) on 12 July 2021. Participation in the program was covered by the Japanese national health insurance system.

### 2.2. Program

The program accommodated up to eight participants per group. At least two psychiatrists or psychologists attended each session. The main facilitators were experienced in CBT for OCD, whereas the co-facilitators were engaged in psychiatric care and had prior experience supporting patients with OCD. Group sessions were generally conducted in a closed format, although participation up to the third session was permitted. To support treatment quality in routine clinical practice, the facilitators received consultation on selected sessions, although it was not provided for every session.

The program consisted of eight weekly group sessions (75 min each), in addition to one individual pre- and post-treatment session. The number of group sessions (eight) was set considering the practical constraints in routine clinical practice, including staff availability. The program was based on the CBT manual for OCD developed by the Ministry of Health, Labour, and Welfare, as well as the manual by Shinmei et al. (2017). The pre-treatment individual session was designed to assess OCD symptoms, provide psychoeducation, and create a draft hierarchy chart in preparation for the group session. The post-treatment individual session, conducted one month after completion of the group sessions, was used to evaluate subsequent symptoms.

The group sessions were primarily structured around exposure and response prevention (ERP), the most widely recommended CBT approach for OCD. Group Session 1 included participant introductions, an explanation of the ERP, and the development of individual anxiety hierarchies. Group sessions 2–7 focused on in-session ERP, with participants assigned weekly ERP homework. Feedback and suggestions were solicited in each session, and strategies were devised to encourage mutual support among participants. Group Session 8 served as a review of prior sessions and addressed relapse prevention. At the conclusion of the final session, the participants exchanged words of encouragement and appreciation (Supplementary Table S1).

### 2.3. Measures

The primary outcomes of this study were feasibility and acceptability. Feasibility was evaluated using the program dropout rate, and assessed based on participants’ feedback regarding program duration. Acceptability was also assessed based on the feedback regarding their willingness to participate in a similar program in the future. Additionally, other themes derived from participants’ free-text responses were descriptively summarized to identify common themes related to feasibility and acceptability. Clinical outcomes were evaluated as secondary outcomes using the Self-Rating Yale–Brown Obsessive Compulsive Scale (Y-BOCS-SR) and other self-report measures.

The Y-BOCS-SR was assessed at four time points: the pre-treatment individual session, the first group session, the last group session, and the post-treatment individual session. These time points were selected to determine changes in OCD symptoms across both the individual and group phases of the program. Other self-report measures were assessed only before and after the group sessions in order to reduce participant burden and maintain feasibility in routine clinical practice. Participants completed and submitted the questionnaire before the start of each session.

#### 2.3.1. Demographic Questionnaire

Sociodemographic data included age, sex, education, marital status, cohabitation, comorbidity, subtypes of OCD, antidepressant medication, age of OCD onset, duration of illness, and past treatment with CBT and other psychotherapies.

#### 2.3.2. Program Feedback

To evaluate participants’ perspectives and identify potential areas for program improvement, a written questionnaire was administered after the final group session. Participants rated the program duration (too long, appropriate, or too short) and their willingness to participate in a similar program in the future (yes or no). In addition, they were invited to provide free-text responses explaining their choices for the duration of the program and offering further feedback on the program (Supplementary File S1).

#### 2.3.3. Self-Rating Yale–Brown Obsessive Compulsive Scale

The Self-Rating Yale–Brown Obsessive Compulsive Scale (Y-BOCS-SR) is a self-reported scale used to assess OCD symptoms (Baer, 2012). It comprises two parts: symptom checklists and severity rating. The severity rating consists of ten items, employing a five-point Likert scale ranging from 0 (“not at all”) to 4 (“extremely”), with higher scores indicating more severe symptoms. A moderate to strong correlation exists between the Y-BOCS-SR and Y-BOCS (Hauschildt et al., 2019). The overall internal consistency of the Japanese version has been reported as α = 0.89, and the clinical group had significantly higher scores compared to the non-clinical group (Hamagaki et al., 1999).

#### 2.3.4. Sheehan Disability Scale

Sheehan Disability Scale (SDS) is a self-reported scale used to assess daily life functions (Sheehan & Sheehan, 2008). The Cronbach’s alpha of the Japanese versions of SDS was 0.84–0.87. The correlation between the SDS and Global Assessment of Functioning was strong, r = −0.89 (Yoshida et al., 2004). The use of SDS was approved by Dr. Sheehan (Sheehan & Sheehan, 2008).

#### 2.3.5. Center for Epidemiologic Studies Depression Scale

The Center for Epidemiologic Studies Depression Scale (CES-D) is a self-report scale used to assess depressive symptoms (Radloff, 1977). CES-D consists of 20 items, rated on a four-point Likert scale ranging from “rarely or none of the time” to “most or all of the time,” with higher scores indicating more severe symptoms. The Japanese version of CES-D was developed (Shima et al., 1985), and its Cronbach’s alpha was 0.92 (Umegaki & Todo, 2017; Nishiyama et al., 2009). The area under the receiver operating characteristic curve of Japanese version of CES-D was 0.96 [95% confidence interval (CI): 0.94, 0.99]. The cut-off score was 19 for screening (Wada et al., 2007).

#### 2.3.6. State-Trait Anxiety Inventory

The State-Trait Anxiety Inventory (STAI) is a self-report scale developed to assess anxiety symptoms. It consists of two subscales that assess state (STAI-State) and trait (STAI-Trait) anxiety (Spielberger et al., 1970). Each subscale consists of 20 items rated on a four-point Likert scale, including 10 positively worded items that were reverse-coded. Previous studies have consistently supported the two-factor structure of the STAI, which reflects a theoretical distinction between state and trait anxiety (Vigneau & Cormier, 2008). Its correlations with the Beck Anxiety Inventory are moderate (state anxiety: 0.52; trait anxiety: 0.44; (Kabacoff et al., 1997). The Japanese version of the STAI has been developed and revised to a new edition (Hidano et al., 2000). Cronbach’s α of Japanese version of the STAI were 0.88 and 0.89 for the state and trait subscales, respectively (Shimizu, 1981).

#### 2.3.7. Clinical Global Impressions Scale

The Clinical Global Impressions Scale (CGI-S) assesses the severity of patients’ illness on a seven-point scale, ranging from 1 (“normal”) to 7 (“extremely ill”); (Guy et al., 1976). The psychometric properties of the CGI-S have been studied in patients with various mental disorders (Leon et al., 1993; Leucht et al., 2006; Zaider et al., 2003). According to a study involving patients with OCD, the Spearman correlation coefficient between the Y-BOCS and CGI-S was ρ = 0.61, indicating a moderate-to-strong correlation (Cervin & Mataix-Cols, 2022). In this study, strong positive Spearman correlations were found between Y-BOCS-SR and CGI-S scores at both first (ρ = 0.76, *p* < 0.001) and eighth (ρ = 0.73, *p* < 0.001) group sessions.

### 2.4. Statistical Analysis

The feasible sample size could not be estimated in advance, as the study relied on data collected during routine clinical practice. Dropouts were defined as (1) absence from more than four group sessions and (2) absence from the final session.

Based on the hypothesis that individuals with higher severity may perceive the treatment period of this program as short, participants were grouped according to their responses regarding treatment duration and the Y-BOCS-SR severity scores at baseline were compared using the Welch two-sample *t*-test. As this was a preliminary feasibility study, the free-text responses were not subjected to formal thematic analysis. Instead, the full text was included and responses to the free-text responses were descriptively summarized and categorized into themes based on their content.

For the preliminary evaluation of OCD symptoms, anxiety, depression, and overall functional impairment, all analyses were conducted using R version 4.5.0 (R Core Team, 2024). Standardized effect sizes (Hedges’ *g*; Hedges & Olkin, 1985) with 95% CIs were calculated for changes between assessment periods from raw data using the effsize package (Torchiano, 2020).

The trajectories of Y-BOCS scores across assessment periods were analyzed using a linear mixed-effects model with maximum likelihood estimation, implemented in the lmerTest package (Kuznetsova et al., 2017). A random intercept was specified for each participant to evaluate within-subject changes over time. The assessment period was modeled as a categorical variable to examine the differences between each assessment point and to conduct post hoc pairwise comparisons among the time points. The baseline (intercept) was set at each individual pre-treatment session. Pairwise comparisons among selected time points (Group Session 1 vs. Group Session 8, pre-treatment individual session vs. Group Session 8, and pre-treatment individual session vs. post-treatment individual session) were conducted using model-based contrasts of estimated marginal means, implemented in the emmeans package (Lenth, 2024), with *p*-values adjusted using Holm’s method. All observed data were included in this analysis. Sensitivity analysis was then performed to assess the robustness of the model, restricted to participants who completed the Y-BOCS-SR at all four time points.

Normality of the STAI–State, CES-D, and SDS distributions was evaluated prior to analysis. Paired *t*-tests were then used to compare scores between Group Session 1 and Group Session 8. CGI-S, treated as an ordinal variable, was analyzed using the Wilcoxon signed-rank test. Missing data were not imputed, and analyses were conducted using complete cases only.

As this study was conducted in a routine clinical setting, no additional statistical adjustments beyond the prespecified analyses were applied to control for potential sources of bias.

### 2.5. Use of Artificial Intelligence Tools

During the preparation of this work the authors used ChatGPT 5 and DeepL to improve the readability and language of the manuscript. When using ChatGPT 5, we included the prompt: “Proofread the following text for an academic paper.” After its use, the authors reviewed and edited the content as required, and take full responsibility for the content of the published article.

## 3. Results

### 3.1. Participant Characteristics and Dropout Rate

**Figure 1:**
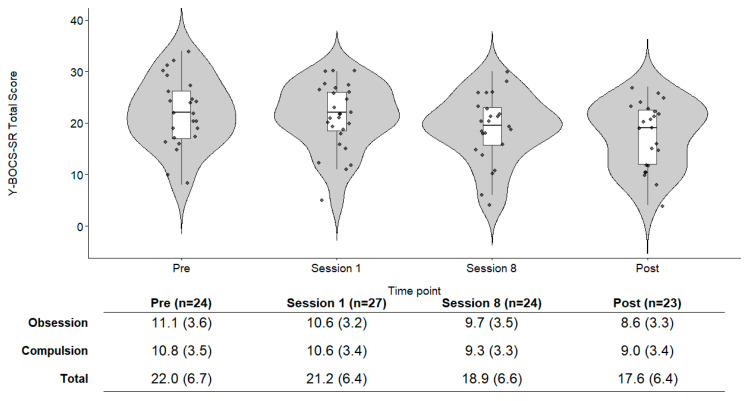
Violin plots and means of Y-BOCS-SR scores across assessment time points. Note. Violin plots illustrate the distribution of Self-Rating Yale–Brown Obsessive Compulsive Scale (Y-BOCS-SR) total scores at four assessment points: the pre-treatment individual session (Pre), the first group session (Session 1), the eighth group session (Session 8), and the post-treatment individual session (Post). The shape of each violin represents the distribution of the observed scores. The embedded boxplots indicate the median and interquartile range, and individual dots represent participant-level observations. The table below the plots presents the mean and standard deviation of Y-BOCS-SR scores at each time point. Cronbach’s α for the Y-BOCS-SR total score was 0.92 at Pre, 0.88 at Session 1, 0.92 at Session 8, and 0.91 at Post. Y-BOCS-SR = Self Rating Yale–Brown Obsessive Compulsive Scale.

**Figure 2:**
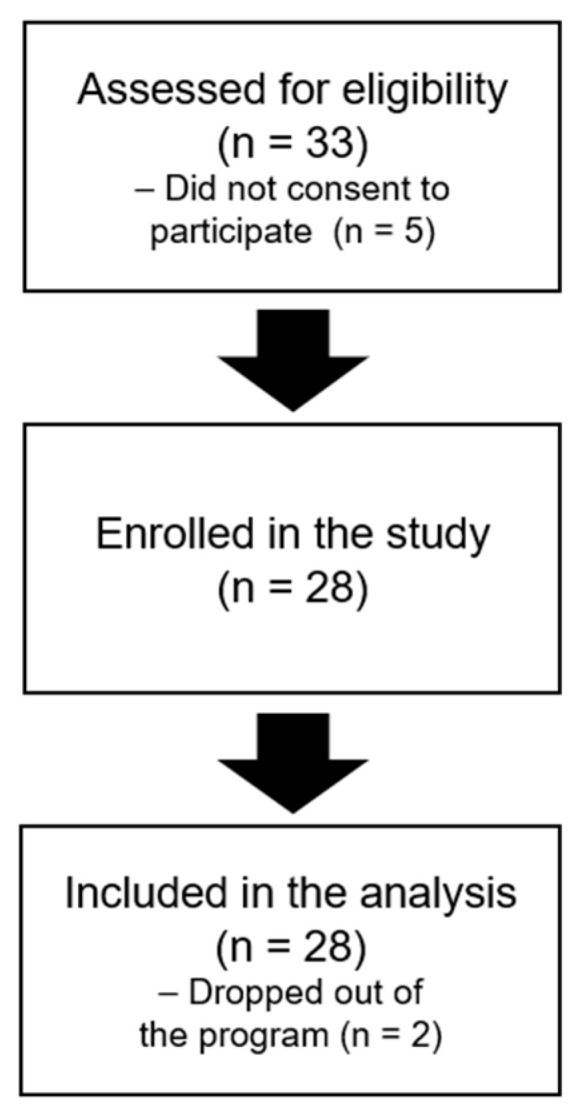
Flow diagram of the study.

Table 1 presents the demographic characteristics of the 28 participants (mean age = 36.1 ± 14.0 years). Twelve (42.9%) participants had comorbid psychiatric disorders, including developmental and mood disorders, and four of them (14.3%) had multiple comorbidities. The most prevalent OCD subtype was contamination (n = 15, 53.6%) followed by checking (n = 7, 25.0%). Baseline of Y-BOCS-SR is shown at Figure 1. Eleven participants (39.3%) were classified as having severe OCD symptoms (Y-BOCS-SR ≥ 24; Goodman et al., 1989). Of these, nine participants were taking antidepressants (selective serotonin reuptake inhibitors or clomipramine), and seven of them had previously received CBT. Two participants (7.1%) dropped out of the program and no other participants missed four or more sessions (Figure 2): one participant experienced difficulty attending outpatient appointments, and the other had severe OCD symptoms, with superstitious obsessions and compulsions as the primary subtype. Due to practical constraints inherent in routine clinical settings, the number of participants attending each session and the number of valid responses varied across time points. Detailed information is provided in Supplementary Table S2.

### 3.2. Feasibility and Acceptability from the Program Feedback

Twenty-four participants completed the questionnaire after Group Session 8, of which 23 responded to the questions regarding the program duration and their interest in future participation in similar programs. Eleven participants provided suggestions for program improvement. As completion of the questionnaire was voluntary, this variation reflected participant dropout, absence from final session, and missing responses. Seven participants (30.4%) reported that the program was too short, 16 (69.6%) considered its duration appropriate, and none reported that it was too long. No significant difference in the baseline Y-BOCS-SR scores was observed between participants who rated the program as too short and those who rated it as appropriate (*t* = 0.79, *p* = 0.45). Nineteen participants (82.6%) answered that they wanted to participate again in similar programs in the future. The themes identified from participants’ open-ended responses are summarized in Table 2. The most frequently mentioned acceptability-related theme was perceived usefulness (n = 13), followed by overall satisfaction (n = 11). For feasibility, program duration (n = 15) and program content (n = 12) were most frequently mentioned. Although no adverse events were reported, some participants commented that they found ERP challenging (n = 2). Additional open-ended program feedback is presented in Supplementary Table S3. For program improvements, several participants expressed a desire for increased interactions among group members and improvements in group composition.

### 3.3. Changes in OCD Symptom Severity

The Y-BOCS-SR scores, assessed prior to the start of each group session, descriptively decreased from 21.2 ± 6.4 at Group Session 1 to 18.9 ± 6.6 at Group Session 8 (Figure 1). With the pre-treatment individual session treated as the baseline, the linear mixed-effects model analysis revealed that OCD symptom severity decreased from pre-treatment individual session to Group Session 8 (estimate = −2.74, *p* = 0.002) and the post-treatment individual session (estimate = −4.16, *p* < 0.001, Supplementary Figure S1). No significant differences were observed between Group Session 1 and 8 (*p* = 0.37) or between Group Session 8 and the post-treatment individual session (*p* = 0.59) based on the post hoc pairwise comparisons; corresponding effect sizes based on raw data were *g* = 0.26, 95% CI [0.06, 0.45] and *g* = 0.20, 95% CI [−0.02, 0.41], respectively. No significant differences were observed between the pre-treatment individual session and Group Session 1 (*p* = 0.94); the corresponding effect sizes based on raw data was *g* = 0.18, 95% CI [−0.44, 0.08]. In contrast, significant reductions were found between the pre-treatment individual session and Group Session 8 (*p* = 0.01), and between the pre- and post-treatment individual sessions (*p* < 0.001); corresponding effect sizes based on raw data were *g* = 0.35, 95% CI [0.01, 0.68] and *g* = 0.62, 95% CI [0.28, 0.95] respectively. In the complete-case analyses (n = 19), OCD symptom severity significantly decreased at the eighth group session (estimate = −2.63, *p* = 0.005) and at the post-treatment individual session (estimate = −4.11, *p* < 0.001), consistent with the findings of the previous analysis. However, it is worth noting that this study minimized patient burden in the clinical setting by eliminating visits and assessments outside of sessions, which resulted in a lacked follow-up data.

### 3.4. Changes in Other Measures

A paired-sample *t*-test revealed a statistically significant reduction in SDS and STAI–State scores between Group Sessions 1 and 8. The effect size (Hedges’ *g*) for the SDS was small (*g* = 0.30, 95% CI [0.08, 0.53]), whereas that for the STAI–State was moderate (*g* = 0.65, 95% CI [0.25, 1.04]). By contrast, no statistically significant changes were observed in the CES-D scores across the same time points (Table 3). Regarding the CGI-S, the median score decreased from 6 to 3, with a significant difference confirmed by the Wilcoxon signed-rank test (*p* = 0.01).

## 4. Discussion

This study evaluated the feasibility and acceptability of hybrid CBT combined individual and group sessions for OCD implemented in routine clinical settings. Unlike interventional trials, there were no incentives, such as compensation, yet the dropout rate was low. None of the participants reported that the duration of the program was too long, although some perceived it as too short. Regarding acceptability, many participants indicated willingness to participate in similar programs in future. In the free-text responses, several participants expressed a desire for increased interactions among group members and for improvements in group composition. Although no changes were observed in the Y-BOCS-SR scores between the first and eighth group sessions, significant reductions were found between the pre-treatment and the eighth group session, as well as between the pre- and post-treatment sessions. While no significant changes were detected in depressive symptoms, functional impairments and overall clinical impressions improved even with group sessions alone.

### 4.1. Dropout and Group Composition

In the current study, the dropout rate was 7.1%, suggesting that the program met one of the indicators of feasibility. Notably, the observed dropout rate was lower than the average dropout rate of 9.8% reported in a previous meta-analysis on CBGT for OCD (Pozza & Dettore, 2017), suggesting that the program performed well even in a routine clinical setting. Although the sample size was relatively small (n = 28), it was larger than those reported in previous studies examining similar hybrid approaches (Kunnari et al., 2024; Taylor & Reeder, 2015). Importantly, as this study aimed to evaluate feasibility in a routine clinical setting, the sample size reflects real-world constraints and is consistent with the exploratory nature of feasibility studies.

Of the two participants who dropped out, one had severe OCD with a primary subtype that differed from that of the others. When symptoms are severe, the progression of symptom improvement may be difficult to discern and may take longer. Furthermore, as noted by Bieling et al. (2022), differences in OCD subtypes may contribute to feelings of isolation in a group setting. This concern was also reflected in participant feedback, in which some individuals expressed a preference for group composition based on severity or subtypes. Taken together, these findings suggest that the future program may benefit from greater attention to group composition, with consideration of feelings of loneliness, particularly regarding OCD subtypes and severity, to enhance treatment adherence.

### 4.2. Implementation Related Challenges from the Program Feedback

Regarding feasibility, several implementation-related challenges were identified. Some participants reported that ERP exercises were challenging or distressing, which may reflect the inherently anxiety-provoking nature of exposure-based interventions. This finding highlights the importance of clearly communicating the therapeutic rationale and providing adequate therapist support during exposure exercises. In this context, the experience and competence of therapists may play an important role in supporting patients during ERP. In the current program, some co-facilitators had limited experience with ERP, emphasizing the importance of establishing training systems to ensure therapist competence and treatment fidelity. In established service models such as the NHS Talking Therapies for anxiety and depression (National Collaborating Centre for Mental Health, 2024), therapists receive structured training and regular supervision, suggesting that the development of similar systematic training frameworks may be beneficial for future implementation. At the same time, developing and integrating such structured training and supervision systems into the Japanese healthcare context remains a key challenge for future implementation.

In routine clinical practice, it was also sometimes difficult for therapists to attend all sessions due to work-related constraints. This issue was reflected in participants’ comments regarding facilitator continuity, and we infer that maintaining consistent staffing may be an important consideration for future implementations of the program.

### 4.3. Acceptability from the Program Feedback

Regarding acceptability, most participants reported that the overall program duration was appropriate, and nineteen participants indicated willingness to participate in a similar program in the future. From descriptive themes, several participants also reported overall satisfaction with the program. However, comments related to program content suggested that some participants felt that sessions progressed too quickly, and several expressed a desire for more time for in-session ERP and greater interaction among participants. These findings suggest that, in addition to the total number of sessions, the duration of individual sessions may warrant further consideration.

### 4.4. Comparison with Previous Interventional Studies Using Y-BOCS-SR

Although the program demonstrated a certain degree of changes in Y-BOCS-SR, the effect size for changes in Y-BOCS-SR scores was smaller than that reported in previous interventional studies of CBGT (Pozza & Dettore, 2017) or effectiveness study of CGBT (Kearns et al., 2010). One reason may be that the program duration and intensity were limited by routine clinical constraints. Previous meta-analytic findings reported that CBGT programs averaged 11 sessions of 120 min each (Jonsson & Hougaard, 2009); thus, the relatively shorter program duration in the present study may have restricted treatment gains. The characteristics of the participants are also likely to have contributed to the effectiveness of the program. Because no exclusion criteria were applied, the sample included individuals with comorbid conditions and severe baseline symptoms, although 71.4% had previously experienced individual CBT either in clinical practice or research. Comorbidities present in this study, such as bipolar disorder and alcohol use disorder, are often excluded from interventional trials. Comorbidities and baseline severity are also associated with the reduction in Y-BOCS-SR.

Additionally, several factors may have contributed to the observed changes in participants, including expectancy effects, natural fluctuations in symptoms, medication changes during the program, non-specific therapeutic effects, measurement points, and therapist competence. Notably, most participants (20 of 28 participants) had a prior history of individual CBT for OCD. Therefore, the present program may have contributed more to the consolidation and maintenance of treatment gains rather than to the reduction in acute symptoms. These findings should therefore be interpreted cautiously, and further studies are needed to specifically clarify the program’s effectiveness.

### 4.5. Potential Contribution of Individual Sessions

The greater pre–post effect size, compared to the group session 1–8 interval, suggests that incorporating at least one individual session may enhance treatment outcomes. Given the heterogeneity of OCD, individualized sessions are valuable for case formulation and treatment personalization, allowing clinicians to address specific symptom profiles and comorbidities that may not be adequately managed in a group format.

However, it remains unclear whether the greater effect observed was attributable to the inclusion of individual sessions or simply to the increased number of total sessions. At the same time, feasibility in routine clinical settings must also be considered, as adding individual sessions increases the demand for clinical resources. Future studies are needed to determine the optimal balance between individualized and group-based components in hybrid CBT programs for OCD.

### 4.6. Limitations

This study had several limitations. First, this was a study with a small sample size and it may have reduced the statistical power to detect modest effects. Second, most participants had previously received individual CBT, raising the possibility that the program functioned more as an augmentation to prior individual treatment than as a standalone group-based program. Third, owing to the observational nature of this study, the potential effects of other concurrent interventions cannot be ruled out. Fourth, since this study was exploratory and a formal qualitative analysis was not performed, the overall interpretation of participants’ feedback should be made with caution. Fifth, symptom severity was assessed before, rather than after group sessions. This assessment schedule was determined by practical constraints in the clinical setting, including limited time following sessions and concerns about participant burden. As a result, both immediate and longer-term follow-up effects of post-individual sessions could not be assessed. Sixth, this study was conducted at a single institution, and the program was run by a small number of therapists. The results were likely influenced by the competencies of the therapists, thereby limiting the generalizability of the findings to other settings or populations. Despite these limitations, the current program may serve as a feasible option for delivering CBT to individuals with OCD in a clinical setting.

## 5. Conclusions

This study preliminarily demonstrated the feasibility and acceptability of a hybrid CBT program for OCD: a CBGT program with individual components implemented as part of routine clinical practice. The low dropout rate and positive feedback from the written questionnaire show the feasibility and acceptability of the program, which was implemented in real-world settings. Future studies with larger samples, extended program duration, and controlled designs are warranted to verify these preliminary findings and clarify the effectiveness of hybrid CBT in routine clinical settings.

## Figures and Tables

**Table 1:** Demographic and Clinical Characteristics of the Participants.

Characteristic (n = 28)	
Age, mean (SD), year	36.1 (14.0)
Gender (male), n (%)	7 (25.0)
Education, n (%)	
University, Graduate School	12 (42.9)
High School	8 (28.6)
Two-year college	6 (21.4)
Middle School	2 (7.1)
Marital status, n (%)	
Married	8 (28.6)
Separated, divorced, widowed	2 (7.1)
Single	18 (64.3)
Cohabitating, n (%)	25 (89.3)
Comorbidity, n (%) *	12 (42.9)
Bipolar Disorder	2 (7.1)
Depression	3 (10.7)
Autism Spectrum Disorder	4 (14.3)
Attention Deficit Hyperactivity Disorder	2 (7.1)
Others **	5 (17.9)
Main Subtype, n (%) *	
Contamination-cleaning	15 (53.6)
Doubting-checking	7 (25.0)
Aggressive	3 (10.7)
Ordering-symmetry	1 (3.6)
Slowness	2 (7.1)
Taking antidepressant medication, n (%)	21 (75.0)
Duration of the illness, mean (SD), year	12.8 (8.3)
Age of onset, mean (SD), year	23.3 (13.0)
Individual CBT for OCD before, n (%)	20 (71.4)
Group CBT for OCD before, n (%)	3 (10.7)
Other psychotherapy before, n (%)	8 (28.6)

Note: * Due to individuals with multiple comorbidities or subtypes, the total number differ from the sum of each category. ** Others include individuals with borderline intellectual functioning, epilepsy, alcohol addiction, eating disorder, and tics.

**Table 2:** Themes identified from open-ended responses regarding the acceptability and feasibility of the program.

Theme	n	Example Comments	
Acceptability			
Perceived usefulness	13	I felt a sense of achievement from having learned a variety of skills through the program.	I could feel a change in myself.
Overall satisfaction	11	It was a very positive experience.	I was very satisfied with the program contents.
Perceived peer support	6	It was encouraging to have people experiencing similar symptoms.	Observing other participants helped me gain an objective perspective.
Group cohesion	4	As the number of sessions decreased, I felt more motivated to continue.	Doing the tasks together with others was very encouraging.
Perceived therapist support	2	I sincerely appreciate your support.	The staff told me that OCD wasn’t my fault but rather “something that was making me act that way”, and that made me feel much better.
Feasibility			
Program duration	15	The program ended just as my mood had started to improve.	Longer sessions might have been exhausting.
Program content	12	I want to review the principles of ERP together once every two sessions.	Each session went by very quickly; I would have liked more time to hear others’ experiences.
Group composition	3	I preferred to have group members with similar symptom subtypes.	Once symptoms improve to some extent, it may be difficult to be in the same group as those with more severe symptoms.
Staffing	2	Increasing the number of staff involved might be beneficial.	I was slightly concerned that some facilitators were not consistently present.
Difficulty of ERP	2	Exposure was painful.	There were some difficult moments.
Attendance	1	I’ve had to take more days off due to personal circumstances.	
Adverse events	0		

Note: Responses to open-ended questions were descriptively categorized into themes related to acceptability and feasibility. Values indicate the number of comments corresponding to each theme. ERP = Exposure and response prevention.

**Table 3:** Difference in mean and effect sizes of CES-D, SDS, STAI-state score.

	Session 1, Mean (SD)	Session 8, Mean (SD)	*df*	Hedges’ *g*	95% Cl	*t*	*p*
CES-D (n = 20)	24.4 (11.3)	24.0 (10.9)	19	−0.05	−0.34, 0.23	0.39	0.70
SDS (n = 22)	16.7 (8.3)	13.0 (7.3)	21	0.30	0.08, 0.53	2.77	0.01 *
STAI-State (n = 18)	55.2 (8.4)	48.7 (10.7)	17	0.65	0.25, 1.04	3.63	0.002 *

Note: Cronbach’s α for the CES-D was 0.90 at Group Session 1 and 0.88 at Group Session 8. Cronbach’s α for the SDS was 0.92 Group Session 1 and 0.88 at Group Session 8. Cronbach’s α for the STAI-state was 0.87 Group Session 1 and 0.93 at Group Session 8. * indicates *p* < 0.05. CES-D = Center for Epidemiologic Studies Depression Scale, SDS = Sheehan Disability Scale, STAI-State = State-Trait Anxiety Inventory-State.

## Data Availability

The data presented in this study are available on request from the corresponding author. The data are not publicly available due to privacy restrictions involving participant consent.

## Supplementary Materials

The following supporting information can be downloaded at: https://www.mdpi.com/article/10.3390/bs16040529/s1, Figure S1: Trajectory of Y-BOCS-SR total scores estimated by the linear mixed model and observed data; File S1: Program feedback questionnaire, Table S1: Program protocol, Table S2: Program attendance and the number of valid responses to the questionnaire at each point in time, Table S3: Open-ended program feedback.

## Abbreviations

The following abbreviations are used in this manuscript:

CBGT	Group cognitive behavioral therapy
CBT	Cognitive behavioral therapy
CES-D	Center for Epidemiologic Studies Depression Scale
CGI-S	Clinical Global Impressions Scale
ERP	Exposure and response prevention
OCD	Obsessive–compulsive disorder
SDS	Sheehan Disability Scale
STAI	State-Trait Anxiety Inventory
Y-BOCS-SR	Self-Rating Yale–Brown Obsessive Compulsive Scale

## References

[B1-behavsci-16-00529] Abramowitz J. S. (2006). The psychological treatment of obsessive—Compulsive disorder. The Canadian Journal of Psychiatry.

[B2-behavsci-16-00529] American Psychiatric Association (2007). American psychiatric association (APA) practice guideline for obsessive-compulsive disorder.

[B3-behavsci-16-00529] Baer L. (2012). Getting control: Overcoming your obsessions and compulsions.

[B4-behavsci-16-00529] Bandelow B., Allgulander C., Baldwin D. S., Costa D., Denys D., Dilbaz N., Domschke K., Hollander E., Kasper S., Möller H. J., Eriksson E., Fineberg N. A., Hättenschwiler J., Kaiya H., Karavaeva T., Katzman M. A., Kim Y. K., Inoue T., Lim L., Zohar J. (2023). World federation of societies of biological psychiatry (WFSBP) guidelines for treatment of anxiety, obsessive-compulsive and posttraumatic stress disorders—Version 3. Part II: OCD and PTSD. The World Journal of Biological Psychiatry.

[B5-behavsci-16-00529] Bieling P. J., McCabe R. E., Antony M. M. (2022). Cognitive-behavioral therapy in groups.

[B6-behavsci-16-00529] Cabedo E., Carrió C., Belloch A. (2018). Stability of treatment gains 10 years after cognitive behavioral therapy for obsessive-compulsive disorder: A study in routine clinical practice. International Journal of Cognitive Therapy.

[B7-behavsci-16-00529] Cervin M., Mataix-Cols D. (2022). Empirical severity benchmarks for obsessive-compulsive disorder across the lifespan. World Psychiatry.

[B8-behavsci-16-00529] Elsner B., Wolfsberger F., Srp J., Windsheimer A., Becker L., Jacobi T., Kathmann N., Reuter B. (2020). Long-term stability of benefits of cognitive behavioral therapy for obsessive compulsive disorder depends on symptom remission during treatment. Clinical Psychology in Europe.

[B9-behavsci-16-00529] Farrell N., Deacon B., McKay D., Storch E. (2013). Therapist barriers to the dissemination of exposure therapy. Handbook of treating variants and complications in anxiety disorders.

[B10-behavsci-16-00529] Friedman-Ezra A., Keydar-Cohen K., van Oppen P., Eikelenboom M., Schruers K., Anholt G. E. (2024). Loneliness in OCD and its determinants. Psychiatry Research.

[B11-behavsci-16-00529] Gellatly J., Pedley R., Molloy C., Butler J., Lovell K., Bee P. (2017). Low intensity interventions for obsessive-compulsive disorder (OCD): A qualitative study of mental health practitioner experiences. BMC Psychiatry.

[B12-behavsci-16-00529] Gillihan S. J., Williams M. T., Malcoun E., Yadin E., Foa E. B. (2012). Common pitfalls in exposure and response prevention (EX/RP) for OCD. Journal of Obsessive-Compulsive and Related Disorders.

[B13-behavsci-16-00529] Goodman W. K., Price L. H., Rasmussen S. A., Mazure C., Fleischmann R. L., Hill C. L., Heninger G. R., Charney D. S. (1989). The yale-brown obsessive compulsive scale: I. development, use, and reliability. Archives of General Psychiatry.

[B14-behavsci-16-00529] Guy W. (1976). ECDEU assessment manual for psychopharmacology.

[B15-behavsci-16-00529] Hamagaki S., Takagi S., Urushihara Y., Ishisaka Y., Matsumoto M. (1999). Development and use of the Japanese version of the self-report Yale-brown obsessive compulsive scale. Seishin Shinkeigaku Zasshi.

[B16-behavsci-16-00529] Hauschildt M., Dar R., Schröder J., Moritz S. (2019). Congruence and discrepancy between self-rated and clinician-rated symptom severity on the Yale–brown obsessive-compulsive scale (Y-BOCS) before and after a low-intensity intervention. Psychiatry Research.

[B17-behavsci-16-00529] Hedges L. V., Olkin I. (1985). Statistical methods for meta-analysis.

[B18-behavsci-16-00529] Hidano T., Fukuhara M., Iwawaki M., Soga S., Spielberger C. D. (2000). State trait anxiety inventory (form JYZ) test manual. (Japanese adaptation of STAI).

[B19-behavsci-16-00529] Igakutushinsha (2025). Quick reference table for medical procedure points: April 2025 supplement.

[B20-behavsci-16-00529] Jonsson H., Hougaard E. (2009). Group cognitive behavioural therapy for obsessive-compulsive disorder: A systematic review and meta-analysis. Acta Psychiatr Scand.

[B21-behavsci-16-00529] Kabacoff R. I., Segal D. L., Hersen M., Van Hasselt V. B. (1997). Psychometric properties and diagnostic utility of the beck anxiety inventory and the state-trait anxiety inventory with older adult psychiatric outpatients. Journal of Anxiety Disorders.

[B22-behavsci-16-00529] Kearns C., Tone Y., Rush G., Lucey J. V. (2010). Effectiveness of group-based cognitive–behavioural therapy in patients with obsessive–compulsive disorder. The Psychiatrist.

[B23-behavsci-16-00529] Kobak K. A., Rock A. L., Greist J. H. (1995). Group behavior therapy for obsessive-compulsive disorder. The Journal for Specialists in Group Work.

[B24-behavsci-16-00529] Kunnari L., Mainz S., Granö N., Ranta K., Mäkelä A., Salkovskis P. (2024). Blended individual and group CBT for OCD in adolescents: Model description and a feasibility study. The Cognitive Behaviour Therapist.

[B25-behavsci-16-00529] Kuznetsova A., Brockhoff P. B., Christensen R. H. B. (2017). lmerTest package: Tests in linear mixed effects models. Journal of Statistical Software.

[B26-behavsci-16-00529] Lenth R. V. (2024). Emmeans: Estimated marginal means, aka least-squares means *(Version 1.10.2). Comprehensive R Archive Network (CRAN)*.

[B27-behavsci-16-00529] Leon A. C., Shear M. K., Klerman G. L., Portera L., Rosenbaum J. F., Goldenberg I. (1993). A comparison of symptom determinants of patient and clinician global ratings in patients with panic disorder and depression. Journal of Clinical Psychopharmacology.

[B28-behavsci-16-00529] Leucht S., Kane J. M., Etschel E., Kissling W., Hamann J., Engel R. R. (2006). Linking the PANSS, BPRS, and CGI: Clinical implications. Neuropsychopharmacology.

[B29-behavsci-16-00529] Mukai K., Kyosuke Y., Ogino S., Hosoi Y., Hayashida K., Matsunaga H. (2025). Benefits and barriers associated with using cognitive-behavioral therapy to treat obsessive-compulsive disorder: A narrative review. Front Psychiatry.

[B30-behavsci-16-00529] Murray C. J. L., Lopez A. D., Murray C. J. L., Lopez A. D., World Health Organization, World Bank, Harvard School of Public Health (1996). The global burden of disease: A comprehensive assessment of mortality and disability from diseases, injuries, and risk factors in 1990 and projected to 2020: Summary.

[B31-behavsci-16-00529] National Collaborating Centre for Mental Health (2024). NHS talking therapies for anxiety and depression manual *(Version 7.1)*.

[B32-behavsci-16-00529] National Institute for Health and Care Excellence (2005). Obsessive-compulsive disorder and body dysmorphic disorder: Treatment: Clinical guideline [NICE guideline CG31].

[B33-behavsci-16-00529] Nishiyama T., Ozaki N., Iwata N. (2009). Practice-based depression screening for psychiatry outpatients: Feasibility comparison of two-types of center for epidemiologic studies depression scales. Psychiatry and Clinical Neurosciences.

[B34-behavsci-16-00529] Pozza A., Dettore D. (2017). Drop-out and efficacy of group versus individual cognitive behavioural therapy: What works best for obsessive-compulsive disorder? A systematic review and meta-analysis of direct comparisons. Psychiatry Research.

[B35-behavsci-16-00529] Radloff L. S. (1977). The CES-D scale: A self-report depression scale for research in the general population. Applied Psychological Measurement.

[B36-behavsci-16-00529] R Core Team (2024). R: A language and environment for statistical computing.

[B37-behavsci-16-00529] Schwartz C., Schlegl S., Kuelz A. K., Voderholzer U. (2013). Treatment-seeking in OCD community cases and psychological treatment actually provided to treatment-seeking patients: A systematic review. Journal of Obsessive-Compulsive and Related Disorders.

[B38-behavsci-16-00529] Sheehan K. H., Sheehan D. V. (2008). Assessing treatment effects in clinical trials with the discan metric of the sheehan disability scale. International Clinical Psychopharmacology.

[B39-behavsci-16-00529] Shima S., Shikano T., Kitamura T., Asai M. (1985). New depressive self-rating scale. Psychiatry.

[B40-behavsci-16-00529] Shimizu H. A. I. (1981). Development of the Japanese edition of the spielberger state-trait anxiety inventory (STAI) for student use. Japanese Journal of Educational Psychology.

[B41-behavsci-16-00529] Shinmei I., Kanie A., Kobayashi Y., Nakayama N., Takagishi Y., Iijima S., Takebayashi Y., Horikoshi M. (2017). Pilot study of exposure and response prevention for Japanese patients with obsessive-compulsive disorder. Journal of Obsessive-Compulsive and Related Disorders.

[B42-behavsci-16-00529] Spielberger C. D., Gorsuch R. L., Lushene R. E. (1970). STAI manual for the state-trait anxiety inventory (“self-evaluation questionnaire”).

[B43-behavsci-16-00529] Taylor R., Reeder C. (2015). Intensive individual and group cognitive behavioural therapy for obsessive-compulsive disorder. American Journal of Psychotherapy.

[B44-behavsci-16-00529] Timpano K. R., Çek D., Rubenstein L. M., Murphy D., Schmidt N. B. (2014). Exploring the association between obsessive-compulsive symptoms and loneliness: Consideration of specificity and gender. Journal of Cognitive Psychotherapy.

[B45-behavsci-16-00529] Torchiano M. (2020). Effsize: Efficient effect size computation *(Version 0.8.1). Comprehensive R Archive Network (CRAN)*.

[B46-behavsci-16-00529] Umegaki Y., Todo N. (2017). Psychometric properties of the Japanese CES-D, SDS, and PHQ-9 depression scales in university students. Psychological Assessment.

[B47-behavsci-16-00529] Vigneau F., Cormier S. (2008). The Factor structure of the state-trait anxiety inventory: An alternative view. Journal of Personality Assessment.

[B48-behavsci-16-00529] Visser H., Megen H. V., Oppen P. V., Hoogendoorn A., Glas G., Neziroglu F., Balkom A. V. (2017). The impact of poor insight on the course of obsessive-compulsive disorder in patients receiving naturalistic treatment. Journal of Obsessive-Compulsive and Related Disorders.

[B49-behavsci-16-00529] Wada K., Tanaka K., Theriault G., Satoh T., Mimura M., Miyaoka H., Aizawa Y. (2007). Validity of the center for epidemiologic studies depression scale as a screening instrument of major depressive disorder among Japanese workers. American Journal of Industrial Medicine.

[B50-behavsci-16-00529] Wang Y., Miguel C., Ciharova M., Amarnath A., Lin J., Zhao R., Toffolo M. B. J., Harrer M., Struijs S. Y., de Wit L. M., Cuijpers P. (2026). Effectiveness and acceptability of cognitive–behavioural therapy delivery formats for obsessive–compulsive disorder: Network meta-analysis. The British Journal of Psychiatry.

[B51-behavsci-16-00529] Wheaton M. G., Gershkovich M., Gallagher T., Foa E. B., Simpson H. B. (2018). Behavioral avoidance predicts treatment outcome with exposure and response prevention for obsessive-compulsive disorder. Depress Anxiety.

[B52-behavsci-16-00529] Yalom I., Leszcz M. (2020). The theory and practice of group psychotherapy.

[B53-behavsci-16-00529] Yoshida T., Otsubo T., Tsuchida H., Wada Y., Kamijima K., Fukui K. (2004). The Japanese version of the sheehan disability scale: Development, reliability and validity. Japanese Journal of Clinical Psychopharmacology.

[B54-behavsci-16-00529] Zaider T. I., Heimberg R. G., Fresco D. M., Schneier F. R., Liebowitz M. R. (2003). Evaluation of the clinical global impression scale among individuals with social anxiety disorder. Psychological Medicine.

