# Supplementary material for: Preliminary Feasibility and Acceptability of a Cognitive Behavioral Therapy Combining Group and Individual Sessions for Obsessive–Compulsive Disorder in Clinical Practice
Source: Behav Sci (Basel). 2026 Apr 1;16(4):529. doi: 10.3390/bs16040529 (PMC13113689; doi:10.3390/bs16040529)
Supplement: Supplementary file 1 [file behavsci-16-00529-s001.zip › Supplementary Table S1.pdf]

Supplementary Table S1

Program Protocol

Session	Context
Pre-treatment individual session	Assess OCD symptoms, provide psychoeducation, and create a draft hierarchy chart in preparation for the group session
Group Session 1	Introductions, an explanation of the ERP, and the development of individual anxiety hierarchies
Group Session 2-7	In-session ERP, with participants assigned weekly ERP homework
Group Session 8	Review of prior sessions and addressed relapse prevention
Post-treatment individual session	Evaluate subsequent symptoms

Note: ERP = Exposure and response prevention.
