# Supplementary material for: Preliminary Feasibility and Acceptability of a Cognitive Behavioral Therapy Combining Group and Individual Sessions for Obsessive–Compulsive Disorder in Clinical Practice
Source: Behav Sci (Basel). 2026 Apr 1;16(4):529. doi: 10.3390/bs16040529 (PMC13113689; doi:10.3390/bs16040529)
Supplement: Supplementary file 1 [file behavsci-16-00529-s001.zip › Supplementary Table S3.pdf]

Supplementary Table S3
Open-ended program feedback
**Reasons for considering the program too short:**
1. I found it difficult to continue ERP independently.
2. The program ended just as my mood had started to improve.
3. I became worried about the program ending just as I started to make progress.
4. My anxiety has decreased considerably, and I wanted to continue for a little longer.
5. As there are sessions for introductions and summaries, it might increase reassurance if those were not counted toward the total and more group ERP sessions could be conducted.
6. As the number of sessions decreased, I felt more motivated to continue and wanted a few more sessions.
7. It's great that the program is available during winter when it's easy to feel down, but I wish it would continue past spring when new lives begin (even if not weekly).
**Reasons for considering the program length appropriate:**
1. Longer sessions might have been exhausting.
2. Longer sessions might not have been feasible due to competing demands such as work.
3. I felt a sense of achievement from having learned a variety of skills through the program.
4. Eight sessions seemed just right.
5. I felt that any more sessions may have been too exhausting.
6. This duration was sufficient for me because I could feel a change in myself.
7. Exposure was painful, and sometimes I wondered how many more sessions I would have to endure.
8. I learned a lot and felt a sense of accomplishment.
9. I was able to change my way of thinking, and some of my symptoms improved.
10. Eight sessions felt neither too many nor too few—it was a comfortable number overall.
11. The program was not burdensome. The time commitment itself felt reasonable and appropriate.
12. Depends on the program contents.
13. I've had to take more days off due to personal circumstances.
**Suggestions for improvement:**
1. I wanted to review the principles of ERP together once every two sessions.

2. I preferred to have group members with similar symptom subtypes.
3. Each session went by very quickly; I would have liked more time to hear others' experiences.
4. I would like to have more opportunities for interaction with other participants.
5. I would have liked to hear more specific coping strategies (e.g., "In this situation, it might help to do this or that").
6. Increasing the number of staff involved might be beneficial.
7. I wanted more ERP exercises incorporated within the group sessions.
8. I was very satisfied with the program contents.
9. It was encouraging to have people experiencing similar symptoms. In addition to ERP, it would be beneficial to have time to talk about our difficulties and coping strategies in daily life. Such conversations motivated me to persist with ERP. During the waiting time, everyone seemed livelier when chatting. Once symptoms improve to some extent, it may be difficult to remain in the same group as those with more severe symptoms.
10. I was slightly concerned that some facilitators were not consistently present.
11. My OCD subtype was mainly checking, especially around the kitchen and fire hazards, so every time I ended up checking for keys or appliances. However, doing the tasks together with others was very encouraging.
**Other feedback:**
1. If there were a support group for patients, I think everyone could continue to improve together.
2. Since many participants seemed to be improving just as the eight sessions ended, I felt a slightly longer program may be needed. I am truly grateful for this opportunity and sincerely appreciate your support.
3. Through group participation, I was able to understand other people's struggles and symptoms, which motivated me to keep trying.
4. I felt fortunate to have been placed in an all-female group.
5. If there are other programs or workshops available, I would like to participate.
6. It was a wonderful program. I was able to change myself.
7. I was able to face my own symptoms.
8. Meeting and interacting with others who also suffer from OCD made me feel stronger. I was happy to develop the feeling that we could overcome this together. The staff told me that it wasn't my fault but rather "something (which I named 'the mean voice')" that was making me act that way, and that made me feel much better.

9. I’m glad that we could all work on ERP with the shared intention of getting better together.
10. It was a stimulating program every time. Although I couldn’t attend a few sessions, I looked forward to it each week. I learned a lot, and it was a very positive experience.
11. Thank you very much! I will continue to do my best encouraged by this experience.
12. I’m glad I was able to participate—thank you.
13. Although there were some difficult moments, I feel this program provided valuable time necessary for my personal growth.
14. I’m glad I took part.
15. Observing other participants helped me gain an objective perspective. Hearing words from people with similar struggles was persuasive and relatable, which made it easier to reflect on my own life and apply what I learned.
16. Thank you.
Note. Open-ended program feedback after Group Session 8. The feedback was translated from Japanese by the authors. Minor edits were made for clarity and readability. ERP = Exposure and Response Prevention, OCD = Obsessive-Compulsive Disorder.
