# Supplementary material for: Preliminary Feasibility and Acceptability of a Cognitive Behavioral Therapy Combining Group and Individual Sessions for Obsessive–Compulsive Disorder in Clinical Practice
Source: Behav Sci (Basel). 2026 Apr 1;16(4):529. doi: 10.3390/bs16040529 (PMC13113689; doi:10.3390/bs16040529)
Supplement: Supplementary file 1 [file behavsci-16-00529-s001.zip › Supplementary File S1.pdf]

Supplementary File S1

Program feedback questionnaire

Q1. Do you have any suggestions for program improvements or requests?

( )

Q2. How do you feel about the duration of the program?

・Too short

・Just right

・Too long

Reason ( )

Q3. Would you like to participate in similar programs in the future?

・Yes

・No

Q4. Please feel free to write any other comments or feedback.

( )
