# Supplementary material for: Preliminary Feasibility and Acceptability of a Cognitive Behavioral Therapy Combining Group and Individual Sessions for Obsessive–Compulsive Disorder in Clinical Practice
Source: Behav Sci (Basel). 2026 Apr 1;16(4):529. doi: 10.3390/bs16040529 (PMC13113689; doi:10.3390/bs16040529)
Supplement: Supplementary file 1 [file behavsci-16-00529-s001.zip › Supplementary Table S2.pdf]

Supplementary Table S2

The number of the program attendance and valid responses to the questionnaire at each point in time

	Pre	Session 1	Session 8	Post	Complete valid responses
Program Attendance	24	27	24	23	19
Y-BOCS-SR	24	27	24	23	19
CES-D	-	26	22	-	20
SDS	-	26	23	-	22
STAI-State	-	24	22	-	18
Written Feedback Questionnaire	-	-	24	-	-
Responses for problem duration	-	-	23	-	-
Responses for willing to participate again	-	-	23	-	-

Note: The discrepancy between the number of program attendees and the number of valid responses for each questionnaire is due to the exclusion of non-responses and duplicate responses. For participants who joined the program partway through, the responses from the first session they attended are counted as part of Session 1's responses. Pre = pre-treatment individual session, Session 1 = first group session, Session 8 = eighth group session, Post = post-treatment individual session. Y-BOCS-SR = Self Rating Yale–Brown Obsessive Compulsive Scale, CES-D = Center for Epidemiologic Studies Depression Scale, SDS = Sheehan Disability Scale, STAI-State = State-Trait Anxiety Inventory-State.
