# Supplementary material for: Preliminary Feasibility and Acceptability of a Cognitive Behavioral Therapy Combining Group and Individual Sessions for Obsessive–Compulsive Disorder in Clinical Practice
Source: Behav Sci (Basel). 2026 Apr 1;16(4):529. doi: 10.3390/bs16040529 (PMC13113689; doi:10.3390/bs16040529)
Supplement: Supplementary file 1 [file behavsci-16-00529-s001.zip › Supplementary Figure S1.pdf]

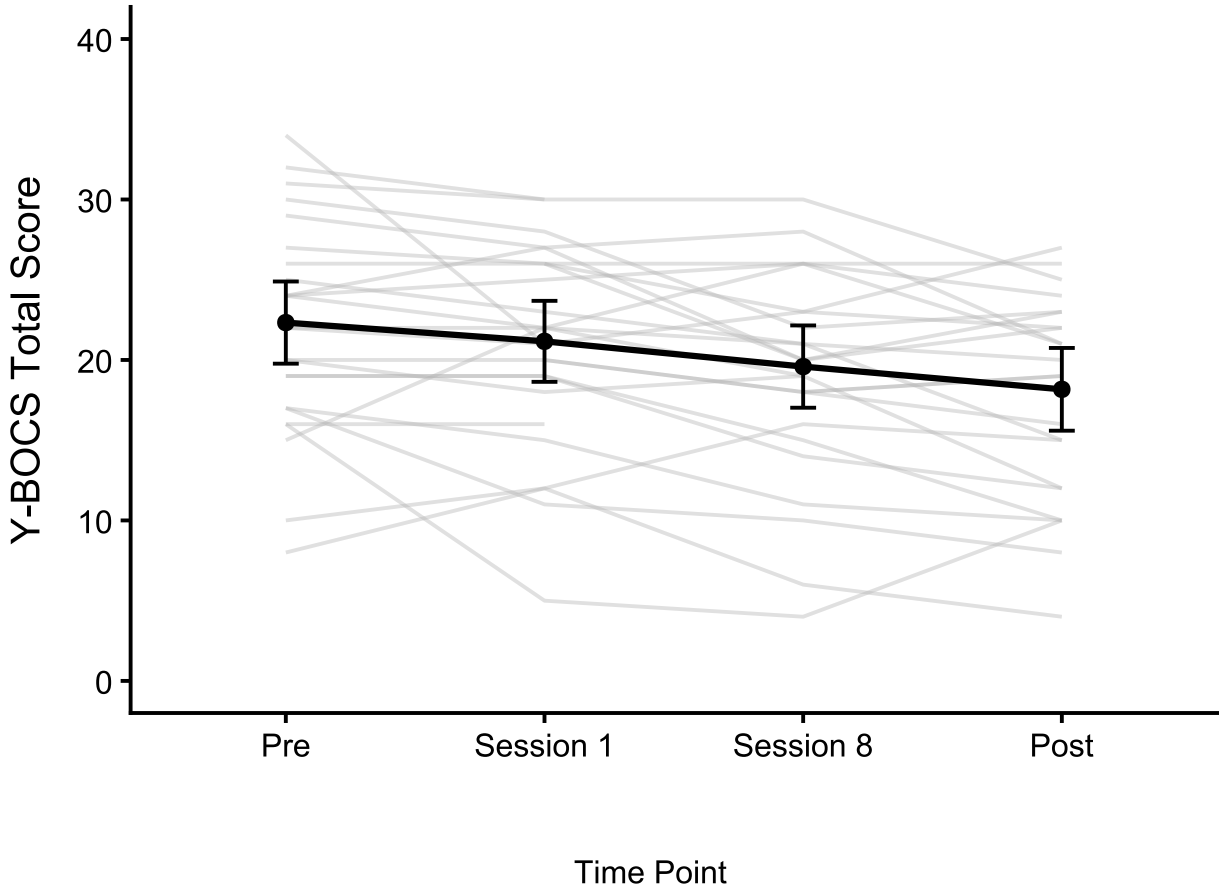

Supplementary Figure S1.
Trajectory of Y-BOCS-SR total scores estimated by the linear mixed model and observed data

Error bars indicate 95% confidence intervals estimated from the linear mixed-effects model, with the pre-treatment individual session treated as the baseline. Thin gray lines represent individual trajectories of Y-BOCS-SR from observed data, and the thick black line indicates model-based estimated means at each time point. Y-BOCS-SR = Self-Rating Yale–Brown Obsessive Compulsive Scale.
